# Short-term influence of Immufen™ on mild allergic rhinitis: a randomized, double-blind, placebo-controlled study

**DOI:** 10.3389/falgy.2024.1390813

**Published:** 2024-10-14

**Authors:** Mamatha K, Manu Kanjoormana Aryan, Prathibha Prabhakaran, Johannah Natinga Mulakal, Syam Das S, Krishnakumar IM, Sreejith Parameswara Panicker

**Affiliations:** ^1^ Department of General Medicine, Divakar’s Specialty Hospital, Bengaluru, India; ^2^ Department of Immunology, Amala Cancer Research Centre, Thrissur, India; ^3^ R&D Centre, Akay Natural Ingredients, Kochi, India; ^4^ Department of Zoology, Advanced Centre for Regenerative Medicine and Stem Cell Research in Cutaneous Biology (AcREM-STEM), University of Kerala, Kariavattom, Thiruvananthapuram, India

**Keywords:** allergic rhinitis, ashwagandha, CurQfen, FenuMat®, immunity, sleep issues, TNSS, BIS

## Abstract

**Introduction:**

Allergic rhinitis (AR) is an IgE-mediated reaction to inhaled allergens, and is a prominent health concern affecting approximately 400 million people worldwide. A comprehensive understanding of AR's pathophysiology is imperative for developing novel therapies, especially considering its frequent co-morbidity with asthma and conjunctivitis. The escalating prevalence of AR is correlated with increased urbanization and environmental pollutants, recognized as prominent contributing factors. Dysregulation in immune networks, Th1/Th2 cytokine imbalance, activation of mast cells and eosinophils are implicated in AR progression. Classic AR symptoms include nasal congestion, nasal itching, rhinorrhea, and sneezing which significantly impact the quality of life, social interactions, and workplace productivity.

**Methods:**

This randomized, double-blind, placebo-controlled, three-arm, three-sequence study was aimed to assess the efficacy of supplementation of a co-delivery form of turmeric extract with ashwagandha extract (CQAB) in comparison with a bioavailable curcumin (CGM) and placebo in alleviating AR symptoms and enhancing the quality of life in individuals with mild AR. Participants received either placebo, CGM, or CQAB twice/day for 28 days, and subjective measures were recorded at the baseline and at the end of study.

**Results:**

CQAB supplementation demonstrated a significant (*P* < 0.05) improvement in Total Nasal Symptom Score (TNSS) compared to placebo and CGM. Furthermore, CQAB administration resulted in enhanced sleep quality (*P* < 0.05) as evaluated by the BIS questionnaire, heightened energy levels, and decreased fatigue and overall mood disturbance (POMS-SF) compared to both placebo and CGM.

**Conclusion:**

The results suggests that CQAB has the potential to be used as a dietary supplement in alleviating AR discomforts.

**Clinical Trial Registration:**

https://ctri.nic.in/Clinicaltrials/login.php; Identifier CTRI/2021/01/030355.

## 1 Introduction

Allergic rhinitis (AR) is a common inflammatory disorder of the upper respiratory tract that has afflicted over 400 million people globally, and its prevalence has increased over the past years (1). It is characterized by symptoms such as sneezing, rhinorrhea, nasal obstruction, itching of the eyes, nose, palate with postnasal drip and cough (2). AR is mostly comorbid with other conditions such as asthma, conjunctivitis and sinusitis which leads to impaired quality of life, cognitive dysfunctions, poor work performance, and poor sleep quality (1, 3–6). Being a chronic disease, AR brings about a substantial financial burden, which involves direct costs for treatment and indirect costs in the form of lower work efficiency (7). It is estimated that the AR impact costs about €1.3 billion in Europe and $20.9 billion in United States (8).

Allergic rhinitis is a IgE-mediated type I hypersensitivity reaction of the nasal mucosa against inhaled allergen (9, 10). The key mechanism in the pathogenesis of AR is the imbalance between the type 1 helper T cells (Th1) and type 2 helper T cells (Th2), as well as in the innate and adaptive immunity which includes antigen-presenting cells, lymphocytes, and T cells (3). On exposure to allergen, the dendritic cells take up the allergen, process, and transport it to draining lymph nodes, which presents it to naïve CD4+ T cells; which in turn are activated and differentiated to Th2 cells. Activated Th2 cells further activates B cells and promotes IgE class switching. This switching promotes B cells to differentiate into plasma cells to produce allergen specific IgE (11), and secretes cytokines, and contributes to vascular permeability, infiltration of eosinophils and other inflammatory cells to nasal mucosa (12–15). IgE in circulation binds to the surface of effector cells such as mast cells and basophils, and leads to degranulation of these cells within the mucosal tissue resulting in the release of mediators and produces the symptoms associated with AR (16).

Management of AR relies on treating the symptoms with antihistamines, nasal/oral glucocorticoids, and nasal decongestants. However, first generation antihistamines like diphenylhydramine and hydrazine are no longer in use due to its adverse effects on central the nervous system (CNS) like sedation, memory impairment, psychomotor dysfunction, and cardiac toxicity (2, 17); on the contrary, second-generation antihistamines are known to penetrate the blood-brain-barrier and cause fewer side effects on the CNS. So, new generation antihistamines like cetrizine, loratadine, desloratadine, fexofenadine, rupatadine, and bilastine are chosen for their efficiency and safety (2). However, they also pose some adverse events like headache, fatigue, somnolence, pharyngitis, dizziness, dry mouth and throat, tachycardia, abdominal distress, and constipation (18–20). Intranasal corticosteroids constitute another approach that has also been reported to pose serious concerns like dryness, burning sensation, blood-tinged secretions, epistaxis, and adverse effects on eyes, bones, and hypothalamic-pituitary-axis (HPA) (21, 22). Allergen-specific immunotherapies are usually introduced under circumstances, where AR conditions are not controlled with pharmacotherapies (1, 23). However, herbal therapy is currently emerging as a safe complementary and alternative medicine to treat and/or manage AR conditions.

Dhanwantari Nighantu, a classical treatise of Ayurveda, has mentioned turmeric for innate host defense and for the treatment of Rhinitis. Ashwagandha on the other hand was recommended for the treatment of congestion (calming cough and difficulty breathing) and to improve body strength, which belong to adaptive immunity. According to the Ayurvedic principles of drug action, both turmeric and ashwagandha possess “Ushna veerya” (hot potency) and the combinations of such herbs can add synergetic pharmacological effects (24, 25). Turmeric and ashwagandha belong to two different groups as per Ayurvedic classics, namely ‘*Katu vipaka*’ and ‘*Madhura vipaka*’ respectively (26). Such Katu-Madhura combinations are preferred for long term use. Moreover, both turmeric and ashwagandha have been considered as safe botanicals for human dose, despite some of their recent reports of hepatotoxicity in patients under polypharmacy (27). Various clinical trials have demonstrated the safety of ashwagandha extracts at 300–1,000 mg/day for up to three months (28). The safety of curcumin has also been demonstrated by a number and human studies with doses ranging from 3,000 to 8,000 mg/day (27, 29, 30). However, diarrhea, headache, rash and yellow stool have been generally reported as the side effects of these botanicals.

Although turmeric and ashwagandha have immunomodulatory properties, their combination has not been subjected to modern scietific studies. Hence, we propose that the simultaneus administration of standardized extracts of turmeric and ashwagandha (Immufen™) with notable water solubility, stability, and bioavailability may have substantial synergistic immunomodulatory benefits on respiratory health and overall life. This paper demonstrates the short-term immunomodulatory effectiveness of Immufen™ against AR illness.

## 2 Materials and methods

### 2.1 Study design, recruitment, and randomization

**Figure 1:**
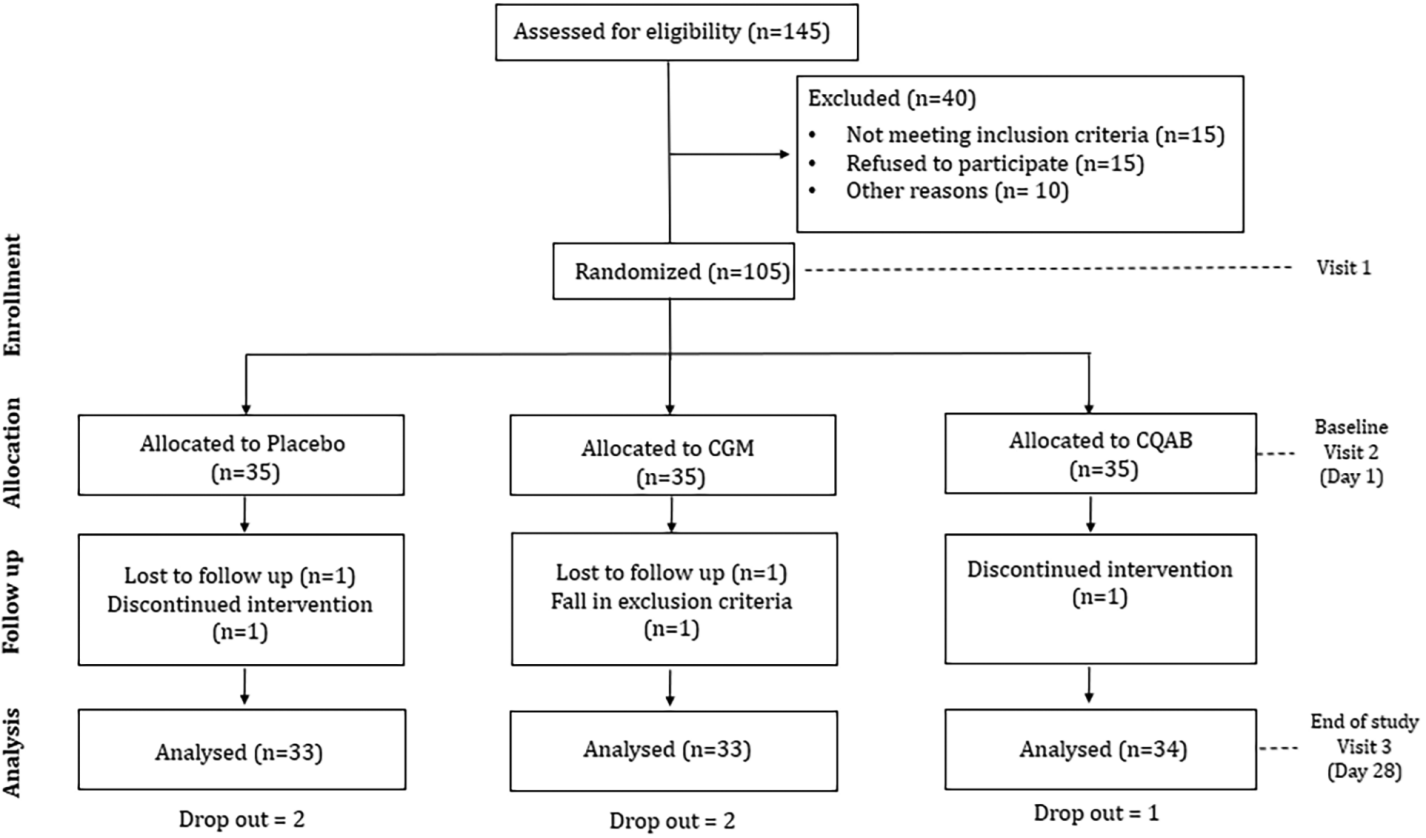
Consort diagram depicting the study design.

The research utilized a randomized, double-blinded, placebo-controlled design with three arms and three sequences, as depicted in the consort diagram (Figure 1). Approval for the study protocol was obtained from the institutional ethical committee at Divakar's Specialty Hospital in Bangalore, India. The protocol was prospectively registered with the clinical trial registry of India (CTRI/2021/01/030355; dated 08/01/2021). Sample size calculation employed G Power Statistical Software (Version 3.1.9.7, Franz Faul University of Kiel, Kiel Germany) based on a previous study on immunity (31). With an assumption of 80% power, 5% significance level, and an anticipated 20% non-compliance/dropout rate, the estimated participant count was 35 per group.

People visiting the outpatient facility at Divakar's specialty hospital were informed about the clinical trial and the interested participants were recruited with screening and informed consent. The screening was based on inclusion/exclusion criteria as detailed in Table 1. Eligible participants were randomly allocated in 1:1:1 ratio into one of the three groups (Placebo, CGM or CQAB) using a computer-generated block randomization technique (www.randomization.com). The participant identification number was assigned on the basis of the order of enrolment in the study. Investigator was provided with randomization codes in separate envelopes by an independent statistician, which ensured the double-blinding efficacy. The interventions were manufactured and packed in identical containers, and labelled similarly.

**Table 1:** Inclusion and exclusion criteria.

Inclusion criteria	Exclusion criteria
Participants of both genders aged between 18 and 45 years.	Individuals requiring prolonged use of inhaled or systemic corticosteroids for asthma in the last 30 days.
Individuals with a history of at least three episodes of allergic rhinitis.	Participants with nasal polyps, chronic respiratory tract infections, and symptoms of viral infections, including COVID-19.
Subjects classified as allergic rhinitis (AR) according to the international guidelines of the American Academy of Allergy Asthma and Immunology.	Those with a recent history of blood donation, immunodeficiency disorders, and any concomitant medication/supplementation affecting the study.
No use of antibiotics within 1 month prior to entering the study or during the study.	Pregnant and lactating women, as well as subjects allergic to herbal products or any component in the test substances.
If female and of childbearing age, willingness to use an acceptable form of birth control, with stability for the past 3 months before baseline and throughout the study.	Participation or recent participation in another clinical trial within the last 3 months prior to the study initiation.
Must be willing and capable of providing informed consent and adhering to the study procedures.	Any additional condition(s) at the Investigator's discretion warranting exclusion from or preventing completion of the study.

### 2.2 Interventions

The interventions used in the study; Placebo, CGM, and CQAB were obtained from Akay Natural Ingredients Cochin, India, and was manufactured using Good Manufacturing Practices (GMP), ensuring identical color, size, and appearance along with a certificate of analysis confirming their food-grade status and material safety data sheet. Material identity was validated through high-performance thin-layer chromatography (HPTLC). Curcuminoids and withanolides content were verified using validated high-performance liquid chromatography (HPLC) methods according to United States Pharmacopeia standards.

Each CGM capsules contained 250 mg CurQfen®-curcumin having 37.5% total curcuminoids (95 ± 5 mg) blended with 150 mg microcrystalline cellulose. Each CQAB included 95 ± 5 mg of curcuminoids and 125 mg AswaBest™-ashwagandha extract formulated with 180 mg of fenugreek mucilage as a Co-delivery form. Placebo comprised 400 mg microcrystalline cellulose, colored to resemble curcumin yellow and flavored with 100 ppm turmeric oil. Participants were instructed to take two capsules daily with breakfast/dinner for 28 days. Adherence was monitored through a pill count strategy, and the blinding effectiveness was assessed by asking participants to predict their allocation.

### 2.3 Outcome measures

The influence of Placebo, CGM and CQAB on severity of nasal symptoms, sleep, and quality of life, was assessed by various questionnaires; TNSS, BIS and POMS-SF.

#### 2.3.1 Total nasal symptom score (TNSS)

The TNSS questionnaire is a validated tool extensively used to gauge the severity of Allergic Rhinitis (AR) symptoms like nasal congestion, runny nose, itching, and sneezing (32). Additionally, it serves as a means to assess the effectiveness of medications for AR (33). Each symptom is rated on a scale from 0 (no symptoms) to 3 (severe), yielding a total score that ranges between 0 and 12. A higher score denotes more severe and pronounced symptoms.

#### 2.3.2 Bergen insomnia scale (BIS)

BIS is a standardized six item questionnaire related to sleep and tiredness over a period of one week/one month (34). It is validated against the widely utilized Pittsburgh Sleep Quality Index (PSQI) (34, 35). The questionnaire encompasses six components: difficulty in initiating and maintaining sleep, early morning awakening, non-restorative sleep, daytime impairment, and overall dissatisfaction with sleep. These aspects are evaluated using an 8-item Likert scale, with a composite score that spans from 0 to 42.

#### 2.3.3 Profile of mood states, short form (POMS-SF)

The POMS-SF is a widely used validated 35-item self-reported questionnaire, which is particularly useful in assessing fatigue-inertia participants (36). It encompasses various subscales such as anger-hostility, confusion-bewilderment, depression-dejection, fatigue-inertia, tension-anxiety, vigor-activity, and friendliness, each rated on a 5-point Likert scale. The standardized scores from the fatigue-inertia and vigor-activity subscales were utilized to monitor changes in symptoms over time, correlating with the quality of life (37). Higher vigor-activity scores indicate positive emotional states, while elevated fatigue-inertia scores indicate poorer health conditions.

#### 2.3.4 Influence of CGM and CQAB on biochemical parameters

Roche-Hitachi Cobas c501 automated biochemical analyser (Manheim, Germany) were used for analysis of biochemical parameters. The clotted blood sample was centrifuged at 3,500 rpm for 10 min at 4℃ to separate serum, which was subsequently stored at −80℃ for biochemical analysis (38). Liver function markers, specifically aspartate aminotransferase (AST) and alanine aminotransferase (ALT), were quantified using standard kit methods provided by M/s Agappe Diagnostics Private Limited in Bangalore, India. Additionally, the concentration of creatinine in the serum sample was determined through the methodology described by Moss et al. (39).

### 2.4 Statistical analysis

Statistical analyses were conducted using IBM's SPSS software version 28. A “*P*” value less than (≤) 0.05 was considered as statistically significant. Data are represented as mean ± standard deviation (SD) for subjective measures, demography, and safety parameters. The planned method of analysis for investigation of treatment effects was Analysis of Covariance (ANCOVA) with post-treatment outcome as the dependent variable, baseline as a covariate and treatment as a between subject's factor. Demographic variables were compared between treatment groups for continuous variables using an independent sample *t*-test.

## 3 Results

### 3.1 Study participants

Detailed baseline demographics of participants are provided in Table 2, showcasing no significant differences between placebo and intervention groups. All participants, aside from experiencing mild seasonal allergic rhinitis discomforts, were found healthy based on baseline medical examinations and analyses of routine biochemical parameters. Out of the 105 enrolled participants (*n* = 35/group), 100 participants completed the 28-day study. Reasons for discontinuation included non-compliance, unexpected emigration, and difficulty adhering to inclusion criteria, with two participants from CGM, one from CQAB, and two from the placebo group affected. Throughout the study, no tolerance issues or adverse events were reported.

**Table 2:** Subject characteristics at baseline and on 28th day.

Parameters	Groups	Day 0	Day 28
Age (years)	Placebo	28 ± 10.15	29 ± 9.89
	CGM	26 ± 10.26	26 ± 10.05
	CQAB	28 ± 9.94	29 ± 9.88
Body weight (kg)	Placebo	69.6 ± 6.39	68.7 ± 4.94
	CGM	69.0 ± 9.07	69.2 ± 6.36
	CQAB	67.4 ± 6.82	68.6 ± 6.23
BMI (kg/m^2^)	Placebo	25.4 ± 2.06	23.12 ± 2.42
	CGM	23.7 ± 2.53	23.95 ± 2.68
	CQAB	24.9 ± 3.14	23.13 ± 2.19
Systolic blood pressure (mmHg)	Placebo	125.0 ± 7.95	122.0 ± 3.70
	CGM	124.0 ± 11.27	122.0 ± 6.76
	CQAB	123.0 ± 10.88	121.0 ± 5.99
Diastolic blood pressure (mmHg)	Placebo	80.0 ± 7.26	84.0 ± 7.19
	CGM	81.0 ± 5.99	84.0 ± 5.64
	CQAB	81.0 ± 7.84	83.0 ± 6.62
Pulse rate (/min)	Placebo	74.0 ± 9.66	74.0 ± 4.41
	CGM	79.0 ± 8.24	74.0 ± 2.09
	CQAB	80.0 ± 9.10	73.0 ± 4.19

BMI, body mass index. Values are expressed as mean ± SD.

### 3.2 Influence of CQAB and CGM on AR symptoms (TNSS)

Analysis of covariance (ANCOVA) for total nasal symptom scores and sub scores are represented in Table 3. Analysis of covariance of CQAB with respect to placebo showed a significant decrease in the symptoms associated with AR; viz., nasal congestion: 34.63% [95% Confidence Interval (CI) (0.74, 1.17); *F* = 12.50; *P* = 0.001], runny nose: 33.01% [95% CI (0.63, 1.06); *F* = 7.50; *P* = 0.008], nasal itching 29.77% [95% CI (0.77, 1.22); *F* = 6.89; *P* = 0.011], sneezing 32.76% [95% CI (0.57, 1.01); *F* = 5.99; *P* = 0.017], and TNSS 31.62% [95% CI (4.64, 5.58); *F* = 50.81; *P* < 0.001]. In contrast with CQAB, CGM administration did not exhibit significant effect on the symptoms and TNSS except for sneezing. The relative changes observed on CGM administration were, nasal congestion: 4.04% [CI (1.19, 1.64); *F* = 0.23; *P* = 0.629], runny nose: 42.92% [CI (0.95, 1.39); *F* = 0.41; *P* = 0.52], nasal itching: 6.39% [CI (1.08, 1.59); *F* = 0.17; *P* = 0.676], sneezing: 30.73% [CI (0.58, 1.04); *F* = 5.10; *P* = 0.027] and in total score of TNSS 8.49% [CI (6.31, 7.38); *F* = 2.80; *P* = 0.099], compared to placebo.

**Table 3:** Influence of CGM and CQAB on AR symptoms (TNSS questionnaire).

Outcome measures	Placebo		CGM		CQAB		Placebo vs. CGM		Placebo vs. CQAB		CQAB vs. CGM	
	Day 0	Day 28	Day 0	Day 28	Day 0	Day 28	*F*-value	*P*-value	*F*-value	*P*-value	*F*-value	*P*-value
Nasal congestion	1.45 ± 0.71	1.48 ± 0.61	1.63 ± 0.89	1.42 ± 0.66	1.61 ± 0.98	0.97 ± 0.62	0.23	0.629	12.50	0.001	8.22	0.006
Runny nose	1.21 ± 0.64	1.27 ± 0.67	1.24 ± 0.83	1.81 ± 0.68	1.35 ± 0.69	0.85 ± 0.55	0.41	0.523	7.50	0.008	7.03	0.010
Nasal itching	1.54 ± 0.71	1.42 ± 0.75	1.45 ± 0.86	1.33 ± 0.73	1.52 ± 0.86	1.00 ± 0.55	0.17	0.676	6.89	0.011	4.50	0.038
Sneezing	1.21 ± 0.64	1.18 ± 0.76	1.30 ± 0.95	0.81 ± 0.52	1.26 ± 0.75	0.79 ± 0.47	5.10	0.027	5.99	0.017	0.04	0.840
Total TNSS	7.60 ± 1.83	7.48 ± 1.43	7.57 ± 1.83	6.84 ± 1.62	7.70 ± 1.50	5.11 ± 1.29	2.80	0.099	50.81	<0.001	24.21	<0.001

TNSS, total nasal symptom score. Values are expressed as mean ± SD. *P* < 0.05 is considered as statistically significant.

Between subject effects of CQAB vs. CGM also showed the significant effect of CQAB in ameliorating symptoms of AR. The percentage improvement observed on administration with CQAB were, nasal congestion: 31.88% [95% CI) (0.75, 1.19); *F* = 8.22; *P* = 0.006], runny nose: 53.13% [95% CI (0.64, 1.02); *F* = 7.03; *P* = 0.010], nasal itching: 24.98% [95% CI (0.77, 1.22); *F* = 4.50; *P* = 0.038], sneezing: 2.93% [95% CI (0.62, 0.96); *F* = 0.04; *P* = 0.840] and an overall score of TNSS: 25.27% [95% CI (4.60, 5.60); *F* = 24.21; *P* < 0.001] compared to CGM (Table 3).

Further comparison of intra (baseline vs. end of study) and inter-group (Placebo vs. CQAB/CGM) analysis using paired and independent “*t*”-test also revealed a similar pattern (Supplementary Table S1). The intragroup analysis of placebo showed no significant effect at the end of study. However, CQAB group showed a significant effect (*P* < 0.05) on both intra and intergroup comparison; while CGM group exhibited significant effect only on the scores of sneezing. All other sub scores associated with AR symptoms as well as the overall total nasal symptom score showed no significant effect (*P* > 0.05) on CGM group.

### 3.3 Influence of CQAB and CGM on sleep quality (BIS questionnaire)

The Analysis of Covariance (ANCOVA) conducted to assess the impact of CQAB in comparison with placebo revealed a significant decrease in BIS score at the study's conclusion, with a reduction of 24.55% [95% CI (10.86, 12.94); *F* = 27.43; *P* < 0.001]. The CGM group demonstrated an 4.79% reduction in BIS score compared to the placebo group [95% CI (13.99, 16.08), *F* = 1.00; *P* = 0.32]. Furthermore, CQAB exhibited a substantial 20.75% reduction in BIS score compared to CGM [95% CI (10.97, 12.85); *F* = 21.51; *P* < 0.001 (Table 4).

**Table 4:** Influence of CGM and CQAB on quality of life (BIS and POMS).

Outcome measures	Placebo		CGM		CQAB		Placebo vs. CGM		Placebo vs. CQAB		CQAB vs. CGM	
	Day 0	Day 28	Day 0	Day 28	Day 0	Day 28	*F*-value	*P*-value	*F*-value	*P*-value	*F*-value	*P*-value
BIS	15.36 ± 3.27	15.78 ± 3.26	15.51 ± 3.01	15.03 ± 2.70	16.00 ± 4.19	11.91 ± 2.72	1.00	0.320	27.43	<0.001	21.51	<0.001
POMS-SF												
Fatigue	10.36 ± 1.93	9.71 ± 1.87	10.09 ± 3.06	8.75 ± 2.33	10.70 ± 1.99	8.44 ± 1.63	16.22	<0.001	30.75	<0.001	0.76	0.385
Vigor	6.42 ± 1.41	7.09 ± 1.94	6.81 ± 2.41	7.63 ± 2.59	6.35 ± 1.93	9.11 ± 3.04	0.90	0.346	10.61	0.002	3.95	0.05
TMD	54.75 ± 4.65	57.12 ± 5.36	55.42 ± 9.02	53.15 ± 7.44	57.23 ± 7.59	40.82 ± 8.44	5.95	0.018	71.50	<0.001	31.80	<0.001

BIS, Bergen insomnia scale; POMS-SF, profile of mood states. Values are expressed as mean ± SD. *P* < 0.05 is considered as statistically significant.

Both intra- and inter-group analyses using *t*-tests underscored the significant improvement in sleep quality as indicated by the BIS score for CQAB, in comparison with baseline, placebo, and CGM (*P* < 0.05). Conversely, both the placebo and CGM groups showed no substantial benefit in sleep (*P* > 0.05) (Supplementary Table S2). These findings highlight the impact of CQAB on improving sleep quality compared to both placebo and CGM, as well as its significant efficacy demonstrated through ANCOVA.

### 3.4 Influence of CQAB and CGM on fatigue, vigor and mood (POMS-SF questionnaire)

At the completion of the study, the POMS-SF analysis revealed a significant improvement in fatigue, vigor, and mood for the CQAB group. ANCOVA comparison of CQAB with placebo indicated a marked decrease in fatigue by 24.91% [95% CI: (7.66, 9.12); *F* = 30.75; *P* < 0.001], a substantial increase in vigor by 28.58% [95% CI (8.24, 9.96); *F* = 10.61; *P* = 0.002], and a notable reduction in mood swings by 28.53% [95% CI (37.77, 43.31); *F* = 71.50; *P* < 0.001]. Conversely, in the CGM group, fatigue decreased by 22.10% [95% CI (7.93, 9.63); *F* = 16.22; *P* < 0.001], vigor increased by 7.70% [95% CI (6.82, 8.44); *F* = 0.90; *P* = 0.346], and mood swings reduced by 6.95% [95% CI (50.99, 55.43); *F* = 5.95; *P* = 0.018] (Table 4).

Further exploration of the CQAB Vs CGM comparison revealed no significant decrease in fatigue: 3.60% [95% CI (7.71, 9.06); *F* = 0.76; *P* = 0.385], but a noteworthy 19.39% increase in vigor [95% CI (8.09, 10.02); *F* = 3.95; *P* = 0.05], and 23.19% decrease in mood disturbances [95% CI (37.74, 43.87); *F* = 31.80; *P* < 0.001] for CQAB (Table 4).

Intra- and inter-group analyses, conducted through *t*-tests, further affirmed the significant beneficial effect of CQAB over CGM, even though CGM also demonstrated notable results when compared to baseline and placebo (Supplementary Table S2). These findings highlight the distinct advantages of CQAB in enhancing mood-related parameters compared to both placebo and CGM.

### 3.5 Influence of CQAB and CGM on clinical safety parameters

Biochemical markers were analyzed employing paired and independent “*t*”-test. Results showed no significant difference (*P* > 0.05) from the baseline or deviations from the normal range at the end of the study (Table 5).

**Table 5:** Influence of placebo, CGM and CQAB on biochemical safety markers.

Parameters	Groups	Day 0	Day 28
AST (U/l)	Placebo	24.43 ± 6.93	30.85 ± 9.65
	CGM	25.87 ± 7.25	31.02 ± 9.29
	CQAB	33.93 ± 14.11	32.11 ± 9.07
ALT (U/l)	Placebo	26.86 ± 11.69	31.92 ± 14.19
	CGM	23.07 ± 13.0	30.79 ± 7.25
	CQAB	35 ± 20.88	30.73 ± 11.12
Serum creatinine (mg/dl)	Placebo	0.88 ± 0.14	0.86 ± 0.11
	CGM	0.86 ± 0.14	0.84 ± 0.15
	CQAB	0.76 ± 0.13	0.85 ± 0.13

ALT, alanine aminotransferase; AST, aspartate aminotransferase. Values are expressed as mean ± SD.

## 4 Discussion

Plant-derived immunomodulators represent a promising area of scientific exploration, with numerous potential candidates already identified for further research and study. Ashwagandha and curcumin are herbal drugs that have been reported to have immunomodulatory effect (40). The active ingredient of ashwagandha, called withanolides and withanolide glycosides, exert its immunomodulatory effect by mobilizing and activating macrophages (41). Curcuminoids, a mixture of curcumin, demethoxy curcumin and bisdemethoxy curcumin commonly referred to as “curcumin”, are the polyphenolic active compounds isolated from turmeric (*Curcuma longa*), and has been used extensively in treating several conditions since ancient times. It has several pharmacological effects and multi-targeting effect including anti-inflammatory, anti-oxidant, anti-amyloidic, anti-cancer, anti-viral, anti-bacterial and anti-fungal properties (42). The ability of curcumin to modulate the immune system depends on its interaction with various immunomodulators like B cells, T cells, dendritic cells, natural killer cells, neutrophils, and macrophages (43). Despite the therapeutic activity of curcumin, poor solubility and bioavailability limits its application. So, in this study, FenuMat® technology (a 100% natural self-emulsifying hydrogel technology based on fenugreek mucilage i.e., galactomannan soluble dietary fibre) was employed to co-deliver curcuminoids and withanolides as a single water-soluble compound (CQAB).

The present randomized, double-blinded, placebo-controlled design with three arms and three sequences was employed to evaluate the short-term immunomodulatory effect of CQAB in ameliorating AR symptoms by balancing the immune response, in otherwise healthy participants with mild AR. The results of the study demonstrated a significant improvement in AR symptoms, quality of life and improved overall sleep quality as evidenced by the TNSS, POMS-SF and BIS questionnaires among the CQAB administered participants compared to CGM and placebo.

AR participant is characterized by bothersome symptoms such as nasal itching, runny nose, eye itching, sneezing, and the most validated tool used to assess these symptoms is TNSS (44). Previous studies have demonstrated that participants exposed to allergen show a change in TNSS, which may be due to the increased influx of inflammatory cells such as eosinophils and basophils (32). Our studies also exhibited an elevated score of nasal congestion, runny nose, nasal itching, sneezing and TNSS. However, CQAB significantly decreased the symptoms associated with AR viz., nasal congestion, runny nose, nasal itching, sneezing and total nasal symptom scores compared to CGM and placebo. CGM group exhibited significant effect only for sneezing.

People with symptoms of AR are often found to be associated with poor social life and sleep quality, which in turn leads to reduced learning ability, productivity at school/work and hence decreased quality of life (45, 46). The results of our study demonstrated a significant improvement in sleep quality when administered with CQAB compared to CGM and placebo. The sleep effect of ashwagandha extracts has already been reported in various clinical trials (47). Recently, curcuminoids were also reported to reduce sleep latency and increase sleep duration in mice (48). Hence it can be postulated that the synergistic effect of ashwagandha and curcumin might have imparted the sleep promoting effect of CQAB.

POMS-SF is a widely used questionnaire for measuring positive and negative moods, and to evaluate mood disturbance (49). Allergies are frequently linked with various symptoms including fatigue, anxiety, irritability, lethargy, depressed mood, and apathy (50). Consistent with this, participants in our study reported elevated levels of mood disturbance, fatigue, and decreased energy at the beginning of the study. However, treatment with CQAB significantly alleviated fatigue, reduced total mood disturbance, and enhanced vigor compared to both the CGM and placebo groups. Previous research on curcumin has demonstrated significant improvements in negative mood, as indicated by the POMS questionnaire (49). Similarly, studies on ashwagandha extract have also reported its ability to mitigate stress, enhance vigor and consequently improving quality of life and workplace performance (51). The absence of adverse events/toxic effects and the observed efficacy in a short span of 28 days further helps to conclude that the supplementation of CQAB may be a safe and easy to use supplement for the management of AR conditions caused by seasonal allergies or food allergens. However, the study has continued for 84 days with a detailed analysis of immunoglobulins, Th1/Th2 cytokines, CD4+/CD8+ cells etc., which will be published elsewhere.

The lack of allergens screening tests such as the skin prick test remains as a major limitation of the present study. Similarly, use of ARIA (Allergic Rhinitis and its Impact on Asthma) guidelines for participants recruitment would have improve the quality of the study. Yet another guidance for the future research would be to compare the efficacy of CQAB with the standard treatment drugs such as oral antihistamines and nasal corticosteroids.

## 5 Conclusion

In summary, our randomized, double-blinded, placebo-controlled, three-arm, three-sequence study using a unique formulation of ashwagandha and turmeric extracts developed with FenuMat® technology (CQAB) demonstrated improved efficacy in alleviating allergic rhinitis symptoms in 28th days, indicating its enhanced immunomodulatory effect. The observation was substantiated by notable reductions in nasal congestion, runny nose, nasal itching, sneezing, and overall total nasal symptom scores compared to both placebo and CGM. However, CGM showed a non-significant improvement in sneezing with respect to CQAB. The alleviation of AR symptoms was further underscored by the improvement in sleep quality among CQAB participants. Furthermore, CQAB administration also led to an overall improvement in the quality of life as evidenced from the decrease in fatigue, increase in vigor, and decrease in overall total mood disturbance compared to placebo and CGM groups. The enhanced efficacy of CQAB over CGM observed in this study may be attributed to the synergistic action of bioavailable curcuminoids with ashwagandha withanolides.

## Data Availability

The original contributions presented in the study are included in the article/Supplementary Material, further inquiries can be directed to the corresponding author.
