# Supplementary material for: Short-term influence of Immufen™ on mild allergic rhinitis: a randomized, double-blind, placebo-controlled study
Source: Front Allergy. 2024 Oct 14;5:1390813. doi: 10.3389/falgy.2024.1390813 (PMC11513368; doi:10.3389/falgy.2024.1390813)
Supplement: Supplementary file 1 [file Table1.docx]

Supplementary Material

**Short-term influence of Immufen™ on mild Allergic Rhinitis: A randomized, double-blind, placebo-controlled study**

**Mamatha K^1^, Manu Kanjoormana Aryan^2^, Prathibha Prabhakaran^3^, Johannah Natinga Mulakal^3^, Syam Das S^3^, Krishnakumar I M^3^, Sreejith Parameswara Panicker^4*^**

^1^Department of General Medicine, Divakar’s Specialty Hospital, Bengaluru, Karnataka, India. [drmamatharameshk@gmail.com](mailto:drmamatharameshk@gmail.com)

^2^Department of Immunology, Amala Cancer Research Centre, Thrissur, Kerala, India. [manu.aryan@amalaims.org](mailto:manu.aryan@amalaims.org)

^3^R&D Centre, Akay Natural Ingredients, Kochi, Kerala, India. [ammuprabhakaran@gmail.com](mailto:ammuprabhakaran@gmail.com), [johannahnatinga.jn@gmail.com](mailto:johannahnatinga.jn@gmail.com), [syam.das@akay-group.com](mailto:syam.das@akay-group.com), [krishnakumar.im@akaybioactives.com](mailto:krishnakumar.im@akaybioactives.com)

^4^Department of Zoology, University of Kerala, Kariavattom, Thiruvananthapuram, Kerala, India. [p.sreejith@gmail.com](mailto:p.sreejith@gmail.com)

***Correspondence:**Sreejith Parameswara Panicker

Department of Zoology,

University of Kerala, Kariavattom P.O,

Thiruvananthapuram, Kerala, 695581, India

e-mail id: [psreejith@keralauniversity.ac.in](mailto:psreejith@keralauniversity.ac.in)

**Table S1.** *P* Values of intra and intergroup analysis of TNSS

| **Outcome measures** | **Placebo** | **CGM** | **CQAB** | **Placebo vs CGM** | **Placebo vs CQAB** | **CQAB vs CGM** |
| --- | --- | --- | --- | --- | --- | --- |
| Nasal congestion | 0.839 | 0.269 | 0.001* | 0.702 | 0.001* | 0.005* |
|  |  |  |  |  |  |  |
| Runny nose | 0.721 | 0.572 | 0.002* | 0.588 | 0.007* | 0.034* |
|  |  |  |  |  |  |  |
| Nasal Itching | 0.458 | 0.513 | 0.005* | 0.621 | 0.010* | 0.039* |
|  |  |  |  |  |  |  |
| Sneezing | 0.861 | 0.011* | 0.009* | 0.029 | 0.015* | 0.845 |
|  |  |  |  |  |  |  |
| Total TNSS | 0.759 | 0.076 | < 0.001* | 0.097 | < 0.001* | < 0.001* |
|  |  |  |  |  |  |  |

*P* < 0.05 are considered as statistically significant which are indicated by ‘*’

**Table S2.** *P* Values of intra and intergroup analysis of BIS and POMS

| **Outcome measures** | **Placebo** | **CGM** | **CQAB** | **Placebo vs CGM** | **Placebo vs CQAB** | **CQAB vs CGM** |
| --- | --- | --- | --- | --- | --- | --- |
| BIS | 0.610 | 0.532 | < 0.001* | 0.309 | < 0.001* | < 0.001* |
|  |  |  |  |  |  |  |
| POMS |  |  |  |  |  |  |
|  |  |  |  |  |  |  |
| Fatigue | 0.088 | 0.034* | < 0.001* | < 0.001* | < 0.001* | 0.522 |
|  |  |  |  |  |  |  |
| Vigor | 0.110 | 0.199 | < 0.001* | 0.337 | 0.002* | 0.036* |
|  |  |  |  |  |  |  |
| TMD | 0.074 | 0.328 | < 0.001* | 0.016* | < 0.001* | < 0.001* |
|  |  |  |  |  |  |  |

BIS: Bergen insomnia scale; POMS: Profile of mood states; TMD: Total mood disturbance. *P* < 0.05 are considered as statistically significant which are indicated by ‘*’
